# Intracardiac Inverse Potential Mapping Using the Method of Fundamental Solutions

**DOI:** 10.3389/fphys.2022.873049

**Published:** 2022-05-16

**Authors:** Shu Meng, Nicholas Sunderland, Judit Chamorro-Servent, Laura R. Bear, Nigel A. Lever, Gregory B. Sands, Ian J. LeGrice, Anne M. Gillis, Jichao Zhao, David M. Budgett, Bruce H. Smaill

**Affiliations:** ^1^ Auckland Bioengineering Institute, University of Auckland, Auckland, New Zealand; ^2^ Bristol Heart Institute, University of Bristol, Bristol, United Kingdom; ^3^ Department of Mathematics, Universitat Autònoma de Barcelona, Bellaterra, Spain; ^4^ IHU Liryc, Electrophysiology and Heart Modeling Institute, Fondation Bordeaux Université, Bordeaux, France; ^5^ Centre de Recherche Cardio-Thoracique de Bordeaux, Univ. Bordeaux, Bordeaux, France; ^6^ Centre de Recherche Cardio-Thoracique de Bordeaux, INSERM, Bordeaux, France; ^7^ Auckland City Hospital, Auckland, New Zealand; ^8^ Department of Medicine, University of Auckland, Auckland, New Zealand; ^9^ Department of Physiology, University of Auckland, Auckland, New Zealand; ^10^ Libin Cardiovascular Research Institute, Calgary University, Calgary, AB, Canada

**Keywords:** atrial arrhythmia, multi-electrode basket catheters, method of fundamental solutions, inverse mapping, endocardial potentials

## Abstract

**Introduction:** Atrial fibrillation (AF) is the most prevalent cardiac dysrhythmia and percutaneous catheter ablation is widely used to treat it. Panoramic mapping with multi-electrode catheters can identify ablation targets in persistent AF, but is limited by poor contact and inadequate coverage.

**Objective:** To investigate the accuracy of inverse mapping of endocardial surface potentials from electrograms sampled with noncontact basket catheters.

**Methods:** Our group has developed a computationally efficient inverse 3D mapping technique using a meshless method that employs the Method of Fundamental Solutions (MFS). An *in-silico* test bed was used to compare ground-truth surface potentials with corresponding inverse maps reconstructed from noncontact potentials sampled with virtual catheters. Ground-truth surface potentials were derived from high-density clinical contact mapping data and computer models.

**Results:** Solutions of the intracardiac potential inverse problem with the MFS are robust, fast and accurate. Endocardial surface potentials can be faithfully reconstructed from noncontact recordings in real-time if the geometry of cardiac surface and the location of electrodes relative to it are known. Larger catheters with appropriate electrode density are needed to resolve complex reentrant atrial rhythms.

**Conclusion:** Real-time panoramic potential mapping is feasible with noncontact intracardiac catheters using the MFS.

**Significance:** Accurate endocardial potential maps can be reconstructed in AF with appropriately designed noncontact multi-electrode catheters.

## Introduction

Accurate identification of regions in the heart which trigger ectopic activation and sustain reentrant arrhythmia is a critical step in effective interventional treatment of heart rhythm disturbance. Sequential contact mapping with catheters introduced percutaneously into one or more heart chambers is widely used for this purpose (Issa et al., 2019), but it can be time-consuming and works poorly in atrial fibrillation (AF) where rhythm is non-stationary. While multi-electrode basket catheters have been used for panoramic mapping in AF (Narayan et al., 2012; Pathik et al., 2018), it is difficult to position them so that the electrodes are uniformly distributed across the atrial surface and in contact with it (Oesterlein et al., 2016; Martinez- Mateu et al., 2018; Pathik et al., 2018).

In principle, inverse methods can be used to reconstruct potentials on the endocardial surface of a cardiac chamber from electrograms recorded at electrodes that are not in contact with it if three-dimensional (3D) chamber geometry is specified, the locations of electrodes are known and the forward problem, which describes the transfer relationship between measured and endocardial surface potentials, is specified accurately. However, the boundary mesh-based solution methods used previously to solve the intracardiac potential inverse problem have shortcomings that are discussed in detail elsewhere (Meng et al., 2017; Meng et al., 2022). With the finite element method (FEM), the transfer matrix is sparse, inherently ill-conditioned and time-consuming to evaluate (Pullan et al., 2005). On the other hand, boundary elements (BEMs) are not robust for measurement points near the heart wall, particularly when surface topology is complex as is commonly the case with the atria. (Pullan et al., 2005).

Meshless methods (MMs) employing the Method of Fundamental Solutions (MFS) (Fairweather and Karageorghis, 1998) have been successfully used to solve the body surface potential inverse problem (Wang and Rudy, 2006). This approach is computationally efficient and robust in a uniform, isotropic domain, an assumption that is realistic for the intracardiac problem. In this case, the MFS provides an inherently simpler representation of the forward problem than boundary mesh-based methods.

While there have been numerous systematic analyses of the efficacy of inverse body surface potential mapping (Ramanathan and Rudy, 2001; Cluitmans et al., 2017; Bear et al., 2018), there have been very few equivalent studies of intracardiac potential mapping (Meng et al., 2022) and no attempt to use the MFS in this setting. In the research reported here, we have used the MFS to address the accuracy with which time-varying potentials on the endocardial surface of the left atrium (LA) can be reconstructed from electrograms recorded inside the chamber with basket catheters where the electrodes may or may not be in contact with the atrial wall. We conclude that accurate real-time panoramic potential mapping and 3D phase mapping are feasible with noncontact intracardiac catheters using the MFS. However, to faithfully recover complex potential fields, such as those seen in AF on the endocardial surfaces of the atria, it is necessary to use catheters that are sufficiently large to capture characteristic features of surface potential variation with an electrode distribution appropriate to resolve it spatially (Meng et al., 2022). We argue that the sampling constraints identified in this study apply to noncontact intracardiac mapping in general.

## Mathematical Background

Noncontact intracardiac potential mapping seeks to reconstruct the potential distribution on the inner surface of a heart chamber from a discrete set of potentials recorded at known points inside the chamber with a multi-electrode catheter. To solve this inverse problem, it is first necessary to formulate the corresponding forward problem. Here, we extend an approach followed by Wang and Rudy (Wang and Rudy, 2006), in which the MFS was applied to inverse body surface potential mapping.

**FIGURE 1:**
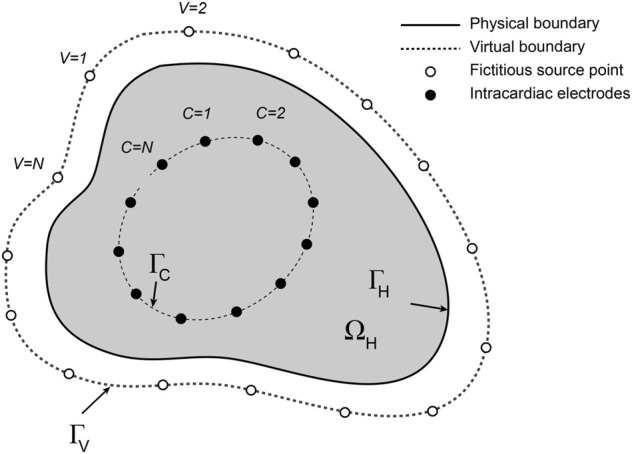
Schematic representation of the intracardiac forward problem. Fictitious electrical sources (open circles) distributed around a virtual boundary ${\text{Γ}}_{V}$ outside the surface ${\text{Γ}}_{H}$ that bounds a heart cavity ${\text{Ω}}_{H}$ generate current flux within the domain. This contributes to potentials recorded with electrodes (closed circles) on a basket catheter. The electrodes lie on the open surface ${\text{Γ}}_{C}$ .

The formulation of the intracardiac forward problem is shown in Figure 1. Potentials $\phi \left(x_{\text{c}}\right)\;$ are recorded at *M* points $x_{\text{c}}$ on the surface *Γ*_*C*_ that bounds the electrodes. A set of *N* fictitious sources is positioned at locations ${\left\{\xi_{i}\right\}}_{i=1}^{N}\;$ along a virtual 3D boundary *Γ*_*V*_ that lies outside the endocardial surface of the cardiac chamber. The linear combination of the Laplace fundamental solution over the sources on ${\left\{\xi_{i}\right\}}_{i=1}^{N}\;$ allows us to have an expression of the potentials in the source free volume Ω_H_ contained in the cardiac chamber. It is assumed that 1) there are no electrical sources or sinks within the heart cavity, 2) conductivity throughout the domain is uniform and isotropic, 3) the electrical properties of the basket catheter can be neglected, and 4) bioelectric processes are quasi-static.

At any instant, the potential ϕ(x) at any point **x** in Ω_H_ due to fictitious sources located on the virtual external boundary $\Gamma_{V}\;\;$ is

$$\phi \left(x\right)=a_{0}+\overset{N}{\underset{i=1}{\sum }}a_{i}G\left(\xi_{i},x\right)$$ (1)

where $a_{0}$ is a constant and $a=\left(a_{1},\ldots ,a_{N}\right)$ is the instantaneous source current at a source ${\left\{\xi_{i}\right\}}_{i=1}^{N}$ . G is the fundamental solution of the Laplace operator in 3D

$$G\left(\xi ,x\right)=\frac{1}{4\pi \left|\xi -x\right|}$$ (2)

and ${\left\{\xi_{i}\right\}}_{i=1}^{N}$ are the 3D locations of the fictious sources and |ξ−x| is the Euclidean distance between **x** and ξ . Note that *a*_*i*_ = *σ* *l*_*i*_ for *i* = 1, *N* where *l*_*i*_ is the source current at *ξ*_*i*_ and *σ* is conductivity.

Potentials at $x_{\text{c}}$ on the surface *Γ*_*C*_ that bounds the electrodes therefore can be estimated by using Eq. 1 for $x\in \Gamma_{c}$ *.* This results in a linear system of equations when they are equated to the measured potentials on the *M* electrodes of the catheter. It should be noted that while the forward problem has been set up here for the continuous surfaces *Γ*_*C*_ and *Γ*_*H*_, this is not a requirement of the MFS.

Solution of this system yields the associated current source densities on the fictitious external boundary $\Gamma_{V}\;$ and corresponding potentials on the endocardial surface *Γ*_*H*_ are then estimated by using Eq. 1 again for $x\in \Gamma_{H}$ *.* This system is inherently under-determined because the number of electrodes *M* is generally less than *N*, the number of fictitious sources needed to map potentials faithfully onto *Γ*_*H*_.

## Methods

**FIGURE 2:**
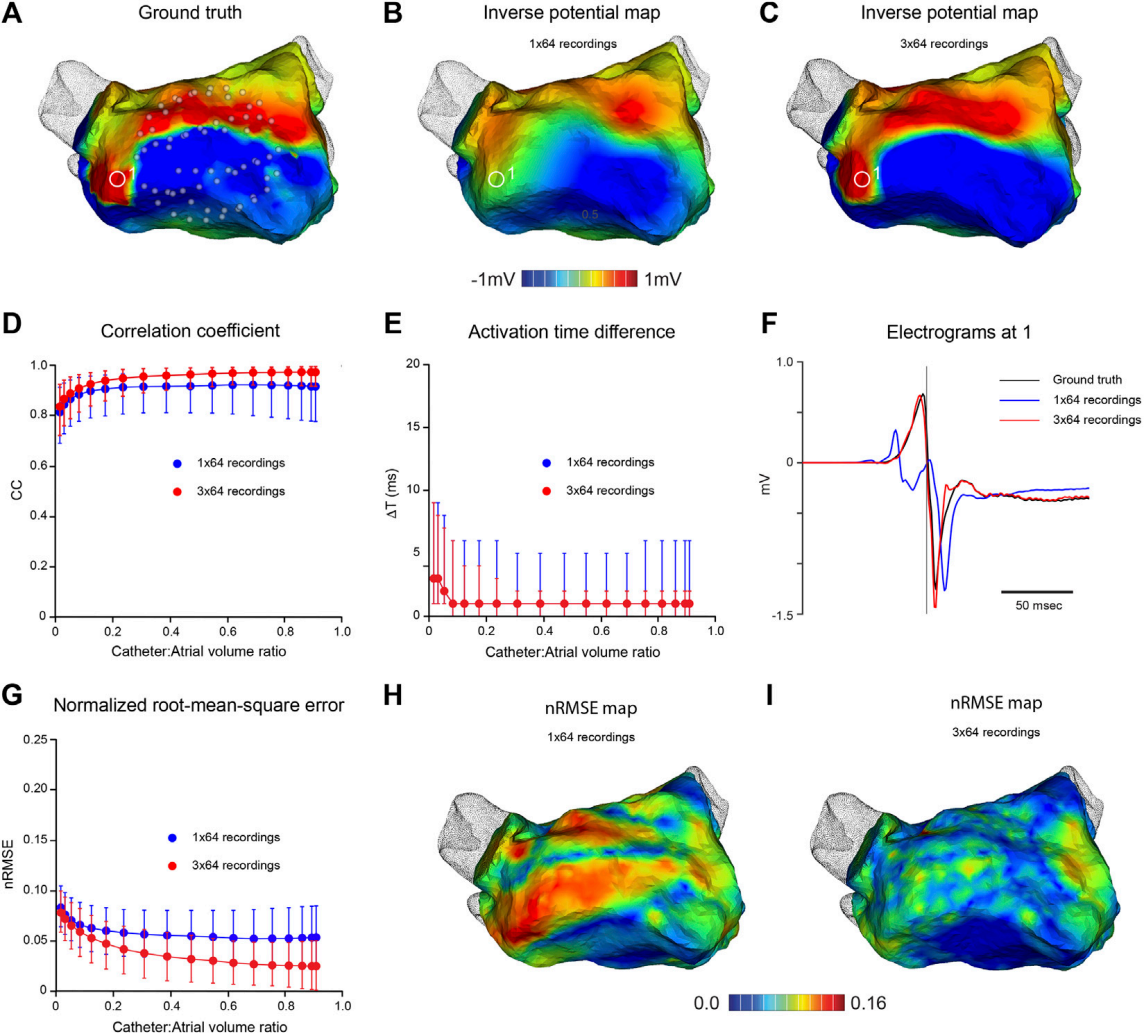
*In silico* analysis of the effects of mapping catheter dimension and electrode distribution on MFS inverse solutions during pacing from distal coronary sinus. Ground-truth LA surface potential distributions during atrial activation are constructed from pace-synchronized contact recordings acquired with a high density electrode array. Corresponding potential fields in the LA cavity are estimated throughout the atrial activation cycle and “sampled” at locations of virtual basket catheter electrodes. Inverse surface potential maps are then reconstructed from these data and compared with ground truth maps. **(A)** Ground-truth LA surface potential distribution at one instant during activation - see red line in **(C)**. Corresponding inverse potential map **(B)** reconstructed from potentials sampled with virtual 64-electrode basket catheter with 0.67 volume fraction relative to LA. Electrodes are distributed uniformly along 8 splines as indicated in **(A)**. **(C)** Ground truth and reconstructed electrograms at 1 in **(A,B)**. In the middle panel, CC **(D)**, nRMSE **(E)** and ΔT **(F)** are presented as functions of relative catheter volume for inverse solutions over one activation cycle. Median values and IQR are given for inverse maps constructed from “recordings” at 64 sites (blue) and 3 sequential “recordings” at 64 sites following stepwise rotation of the catheter through 15° around its axis (red). Both maps in the lower panel relate to the same time as in **(A,B)**. In **(G)**, the distribution of nRMSE on the LA surface is compared with spline location for a potential map constructed from 64 full or near-contact “recordings”, while **(H)** corresponds to **(B)**. Here, the inverse map was constructed from 3 sequential “recordings” at 64 sites with a relative catheter volume fraction of 0.67. **(I)** Ground truth and inverse electrograms at 2) in **(H)**. Abbreviations: MFS, Method of Fundamental Solutions; LA, left atrium; CC, correlation coefficient; nRMSE, normalized root-mean-squared error; ΔT, activation time difference; IQR, interquartile range.

An established computational approach (Ramanathan and Rudy, 2001) was used to quantify the accuracy of inverse potential mapping and key steps are illustrated in Figure 2. First, ground-truth potential distributions were specified on the endocardial surface of the left atrium (LA). The associated field throughout the LA was determined by numerical solution of Laplace’s equation and potentials were sampled at points corresponding to electrode locations on open basket catheters with different electrode distributions across a range of dimensions (Figure 2A). Endocardial surface potentials were then reconstructed from the sampled potentials using the MFS (Figure 2B) and compared with ground-truth potential distributions. This process was continued through a complete atrial activation cycle for stationary rhythms or over several cycles of reentrant activity. Ground-truth electrograms were also compared with corresponding inverse estimates at points across the endocardial surface (Figure 2F) to assess the accuracy with which local activation timing information is reconstructed.

Clinical ground truth data used in this study were acquired as follows. CT imaging was performed in one patient undergoing catheter ablation to treat atrial flutter. The patient gave written informed consent and the study protocol was approved by the Melbourne Health Research and Human Ethics Committee. LA geometry was segmented with the Ensite Verismo^TM^ tool and registered with respect to the mapping system (Ensite Precision, Abbott). A decapolar pacing catheter was positioned in the coronary sinus (CS). A 20-pole Lasso^TM^ catheter was introduced into the LA *via* trans-septal puncture and used to collect 3,200 time-referenced, spatially-registered contact unipolar electrograms across the LA during pacing (300 ms interval) from the distal CS at a sampling rate of 1 kHz. The LA shell was refined to 5,000 vertices and potentials were interpolated by Dirichlet energy minimization (Botsch et al., 2010).

Ground truth data representing polymorphic reentrant atrial activation were simulated. Atrial surface geometry was reconstructed in an anaesthetized sheep (crossbred female, 53 Kg). All procedures were approved by the Animal Ethics Committee of the University of Auckland and conform to the Guide for the Care and Use of Laboratory Animals (National Institutes of Health publication no. 85-23). Gadolinium-enhanced ECG-gated MRI images of the atria (1.0 × 1.0 mm^2^ in-plane resolution approximately parallel to the atrio-ventricular valve plane and 1.6 mm between slices) were acquired with a 3T Siemens Magnetom Skyra^TM^ scanner. LA endocardial surface geometry was segmented using Amira (Thermo Fisher Scientific) and a 3D triangular surface mesh (1,529 nodes) was fitted to the LA with pulmonary veins and left atrial appendage truncated. Ground-truth potential distributions representing polymorphic reentrant atrial activation were modeled on this geometry as follows. Meandering spiral wave reentry was simulated on an isotropic 2D monodomain with Fenton Karma activation kinetics (Fenton and Karma, 1998) using a standard cross-field S1-S2 stimulus protocol (Pandit et al., 2005). Points on the 2D domain were sampled and mapped onto the 3D surface mesh so that surface area was similar in both, with a contour adjacent to the boundary in the former assigned to the mitral valve orifice. Extracellular potentials were approximated from the transmembrane currents computed at each 3D point at a sampling rate of 1 kHz (Supplementary Video S1—Simulated ground truth data—in the Supplementary Material).

The open-source software environment SCIRun (http://www.sci.utah.edu/cibc-software/scirun.html) was used for FEM solutions of the 3D forward problem. (Burton et al., 2011). Intracardiac potential fields were computed from the ground-truth surface potential distributions at successive time instants by solving Laplace’s equation throughout $\;\Omega_{H}$ . The intracardiac field was sampled at points corresponding to electrodes on two basket catheter configurations with 1) 64 channels with 8 equally spaced electrodes along 8 splines at equal radial angles, and 2) 130 channels with 8 equally spaced electrodes along 16 splines at equal radial angles and electrodes at upper and lower poles. Basket dimensions were uniformly scaled to vary catheter:atrial volume ratio. The centroids of catheters and the LA chamber were aligned to allow maximum catheter expansion and to ensure reproducibility between results. Noise was imposed by adding Gaussian noise independently to the electrograms recorded at each electrode with power set at realistic levels. Signal-to-noise ratio (SNR) is quantified as the ratio of root-mean-squared (RMS) voltages of reconstructed electrograms and noise.

Inverse solutions with the MFS were run with purpose-written code. The fictitious boundary was formed by uniform scaling of the atrial surface mesh and sources were associated with each node. Inflation was quantified as the relative volume difference between *Γ*_*V*_ and *Γ*_*H*_ *.* Solutions were stable across the inflation range 2–10% (Supplementary Figure S1) and the value 6% was selected as optimal in the results presented here. Inverse endocardial potential distributions for intracardiac potentials “sampled” with virtual catheters were obtained using zero-order Tikhonov regularization (Tikhonov and Arsenin, 1977) employing the L-curve method to calculate the regularization parameter (Hansen, 2010).

Phase maps were constructed using the approach outlined by Kuklik et al. (Kuklik et al., 2017) Sinusoidal recomposition was applied to electrograms at each LA surface node and the Hilbert transformation was then used to estimate instantaneous phase at these points.

Correspondence between ground-truth and reconstructed potential maps were quantified by evaluating normalized RMS error (nRMSE) and correlation coefficient (CC).

$$nRMSE=\;\sqrt{\frac{\sum_{i=1}^{N}{\left(\emptyset_{GT}^{i}-\;\emptyset_{R}^{i}\right)}^{2}}{\sum_{i=1}^{N}{\left(\emptyset_{GT}^{i}\right)}^{2}}}\;\mathrm{and}\;\;CC=\;\frac{\sum_{i=1}^{N}\left(\emptyset_{GT}^{i}-\;\mu_{GT}\right)\sum_{i=1}^{N}\left(\emptyset_{R}^{i}-\;\mu_{R}\right)}{\sqrt{{\sum_{i=1}^{N}\left(\emptyset_{GT}^{i}-\;\mu_{GT}\right)}^{2}}\sqrt{{\sum_{i=1}^{N}\left(\emptyset_{R}^{i}-\;\mu_{R}\right)}^{2}}}$$ (3)

where *N* is the number of surface points compared, $\emptyset_{GT}^{i}$ and $\emptyset_{R}^{i}$ are ground-truth and reconstructed potentials at surface point *i*, and $\mu_{GT}\;$ and $\mu_{R}$ are mean values for ground-truth and reconstructed potentials, respectively, across the surface.

Activation times (ATs) for ground-truth (AT_GT_) and reconstructed electrograms (AT_R_) were estimated as maximum negative rate of potential change and the activation time difference ΔT at each surface point was evaluated as

$$\Delta T=\left|AT_{GT}-AT_{R}\right|$$ (4)

Programs were written in C or in the MATLAB programming language (The Mathworks, Natick, Massachusetts).

## Results

### Inverse Potential Mapping in Stationary Rhythm

Figure 2 indicates the accuracy with which high-density potential maps can be reconstructed from clinical ground truth electrograms recorded during the relatively uniform spread of LA activation in coronary sinus pacing. Key features of activation were reconstructed from intra-atrial potentials sampled with a virtual 64-electrode noncontact catheter that occupied ∼67% of atrial cavity volume (Figure 2A). However, neither high resolution features of instantaneous potential maps (Figure 2B) nor high frequency components of regional electrograms (Figure 2F) were captured faithfully. Despite this, accuracy measures were high and surprisingly stable across a wide range of catheter dimensions with median CC = 0.92, nRMSE = 0.054 and ΔT = 1 ms for catheter:atrial volume ratios >0.3 with a 64-electrode basket catheter (Figures 2D–F). None of these measures improved with full or near contact between electrodes and endocardial surface, and nRMSE was highest between splines where the spacing of adjacent electrodes was greatest (Figure 2H), indicating that spatial distribution of electrodes on the surface that bounds the catheter is the problem here. Sampling density was increased by moving the catheter and synchronizing the electrograms acquired. The instantaneous potential map in Figure 2C was reconstructed from 192 electrograms “recorded” in 3 sequential steps by rotating the virtual 64-electrode catheter (relative volume ratio 0.67) around its axis in increments of 15°. This markedly improved the match between high-density ground truth and inverse maps (compare Figures 2A,C). The nRMSE between electrodes was substantially reduced (compare Figures 2H,I) and high frequency components of complex local electrograms were reconstructed accurately (Figure 2F). Median CC increased to ∼0.97 and median nRMSE halved across a wide range of catheter dimensions (Figure 2G).

### Inverse Potential Mapping in Reentrant Rhythm

**FIGURE 3:**
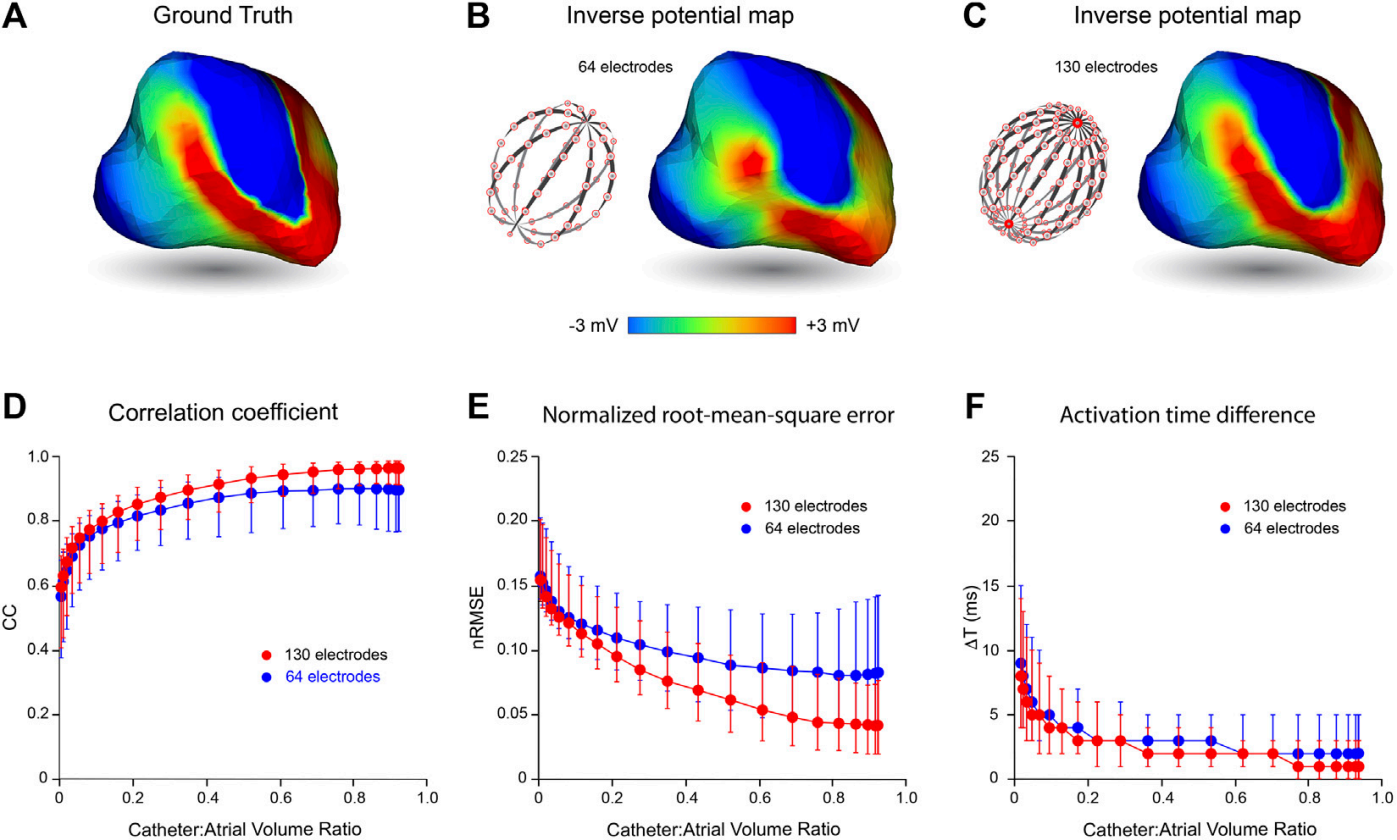
Effects of mapping catheter electrode distribution on inverse solutions with MFS during macro reentry. LA surface potentials throughout 3 activation cycles in simulated atrial flutter are reconstructed from electrograms sampled inside the LA cavity with 64- and 130-electrode basket catheters and compared with ground-truth surface potentials. The upper panel presents typical results for catheters that bound a volume fraction of 0.67 relative to LA volume. These include **(A)** the ground-truth surface potential distribution at one instant during reentrant activation and corresponding potential maps reconstructed using electrograms sampled with **(B)** a 64- electrode basket catheter, and **(C)** a 130-electrode basket catheter. In the lower panel CC **(D)**, nRMSE **(E)** and ΔT **(F)** are presented as functions of relative catheter volume for the 64-electrode catheter (blue) and 130-electrode catheter (red). Median values and IQR are given. Abbreviations: MFS, Method of Fundamental Solutions; LA, left atrium; CC, correlation coefficient; nRMSE, normalized root-mean-squared error; ΔT, activation time difference; IQR, interquartile range.

In Figure 3, we present the effects of catheter dimension and electrode distribution on inverse solutions obtained with the MFS in simulated macroscopic reentrant activation of the LA that replicates features of atrial flutter. Catheter designs considered are a 64-electrode catheter with 8 uniformly spaced electrodes along 8 splines and a 130-electrode catheter that has 8 uniformly distributed electrodes on 16 splines with 2 additional polar electrodes. Once again, the centroids of catheters and LA chamber were aligned.

In the upper panel of Figure 3, an instantaneous ground-truth potential map (Figure 3A) is compared with corresponding inverse maps constructed from potentials sampled with a 64-electrode catheter (Figure 3B) and a 130-electrode catheter (Figure 3C). Endocardial potentials were poorly reconstructed in some regions of the LA with an 8 spline 64-electrode basket catheter but recovered more faithfully with a 16 spline 130-electrode catheter where electrode distribution is more uniform, spatially. As might be expected, errors with the 64-electrode catheter were greatest between splines near the equator where inter-electrode spacing was largest.

The correspondence between ground-truth and reconstructed surface electrograms was quantified for these two catheters over 3 consecutive activation cycles for a range of catheter dimensions, and results are presented in the lower panel of Figure 3. The accuracy with which unanchored reentrant rhythm could be reconstructed was consistently less than for more stable paced rhythms (compare Figure 3 with Figure 2) and it was affected more markedly by relative catheter dimensions. For each of the 3 metrics considered, performance was better at all catheter dimensions with 130-electrode catheters than with the 64-electrode catheters. For example, with 130-electrode catheters, CC approached a median of 0.97 [IQR 0.07] as catheter dimensions were increased, compared with corresponding values around 0.9 [IQR 0.19] with 64-electrode catheters (Figure 3D). Consistent with these results, nRMSE was reduced with increased catheter dimension reaching a median of 0.042 [IQR 0.055] for the 130-electrode catheter compared with 0.083 [IQR 0.099] for 64-electrode catheters (Figure 3E). Finally, ΔT was reduced to a median of 1 ms with a 130-electrode catheter compared with 2 ms for 64-electrode catheters (Figure 3F). All three metrics were relatively stable for catheter volumes >0.6 relative to LA volume.

### Effects of Noise on Inverse Potential Mapping

**FIGURE 4:**
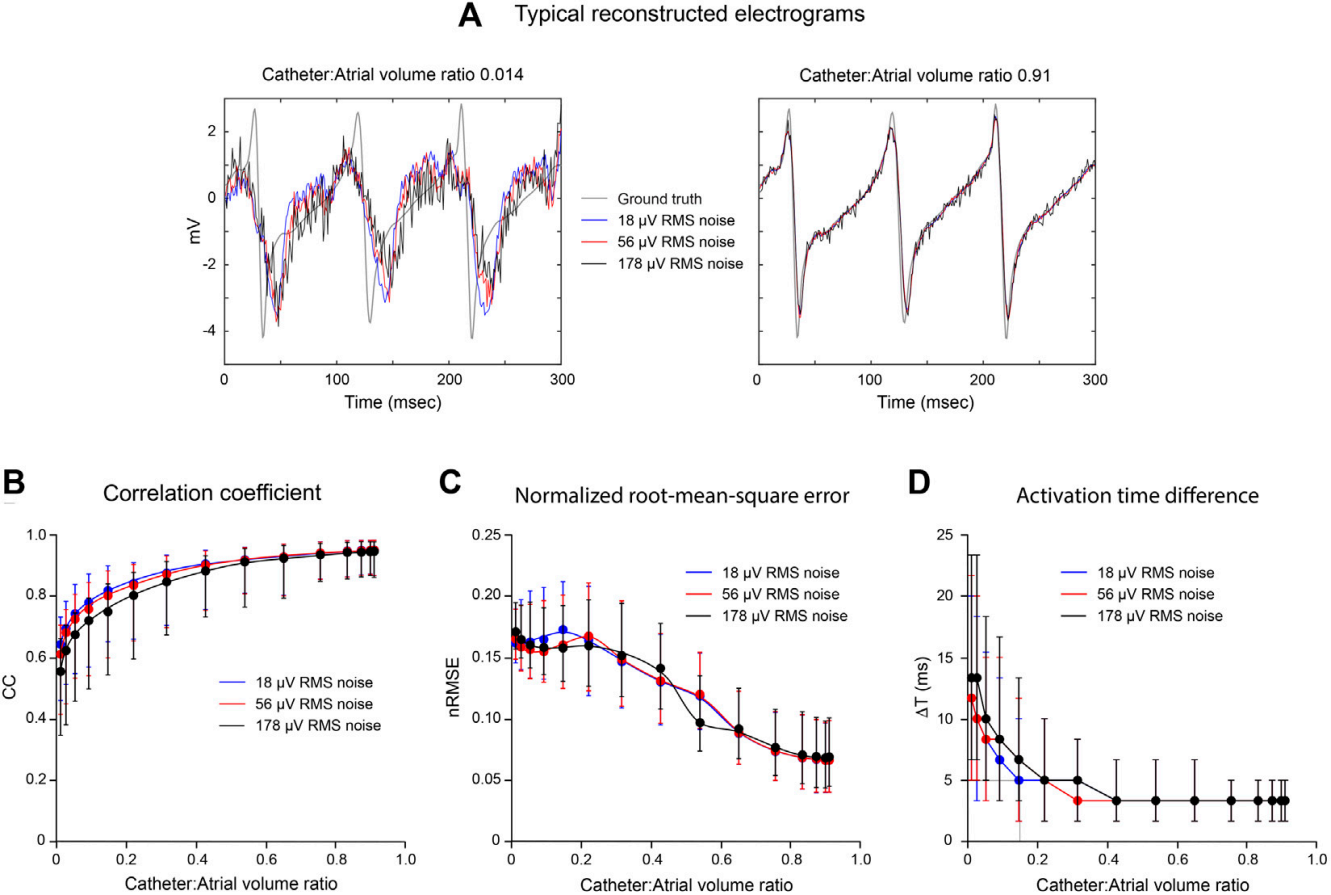
Effects of noise on inverse solutions with the MFS during macro reentry. LA surface potentials throughout activation cycle in simulated atrial flutter are reconstructed from electrograms sampled inside the LA cavity using 130-electrode basket catheters with added Gaussian noise at different mean power levels. All data are compared with ground-truth endocardial potentials. In the upper panel **(A)** electrograms reconstructed from records acquired from a very small and a large basket catheter with 18, 56 and 178 μV of added noise are presented with the corresponding ground-truth electrogram. Catheter volumes relative to LA volume are 0.014 and 0.91, respectively. In the lower panel, CC **(B)**, nRMSE **(C)** and ΔT **(D)** are presented as functions of relative catheter volume for the three noise levels. Median values and IQR are given. Abbreviations: LA, left atrium; CC, correlation coefficient; nRMSE, normalized root-mean-squared error; ΔT, activation time difference.

The effects of noise on the accuracy of inverse endocardial potentials reconstructed with the MFS are summarized in Figure 4. Intra-atrial electrograms were “sampled” with 130-channel catheters during simulated macro-reentry with superimposed Gaussian noise at RMS voltages of 18, 56 and 178 µV. In general, addition of noise had little effect for catheter: LA volume ratios >0.5. However, inverse solutions were progressively degraded by noise at catheter volumes less than this (Figures 4B–D). Comparison of the representative electrograms in Figure 4A provides further insight into this finding. While SNR in reconstructed electrograms scales inversely with added noise, it is much worse for the smaller of the two catheters (6.54, 4.23 and 1.91 for RMS noise voltages of 18, 56 and 178 μV, respectively, compared with 69.56, 22.85 and 6.83 for the larger catheter). It is also noteworthy that while our inverse solutions do not recover higher frequency components in the ground truth electrograms when the catheter is small this is not systematically altered by noise.

### Inverse Phase Mapping in Macroscopic Reentry

**FIGURE 5:**
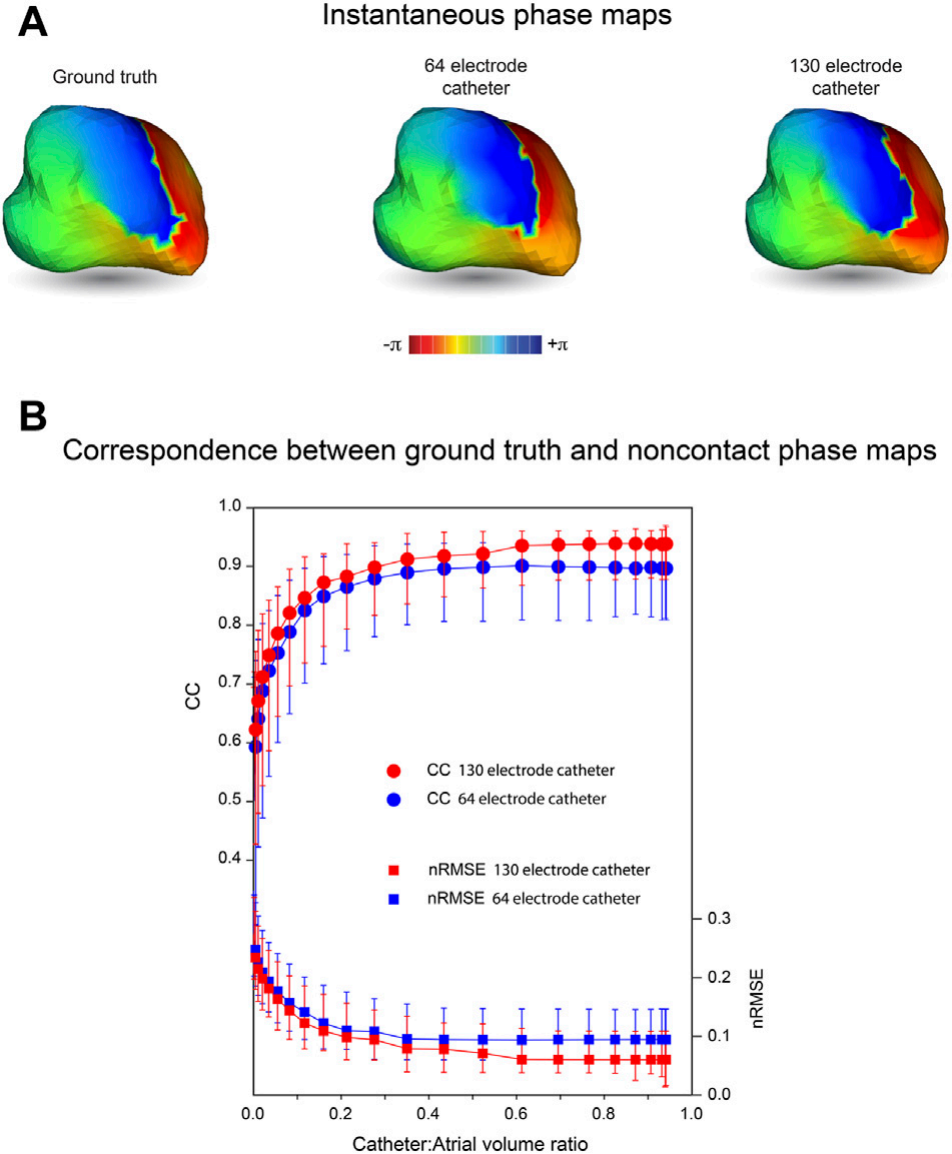
Phase maps of inverse solutions with MFS during macro-reentry. LA surface potentials throughout activation cycle in simulated atrial flutter are reconstructed from electrograms sampled inside the LA cavity using 64- and 130-electrode basket catheters. The centrally located catheters occupy 67% of LA volume. **(A)** Ground truth phase map at one instant during activation compared with corresponding phase maps for surface potentials reconstructed from electrograms recorded with 64-electrode and 130-electrode basket catheters. **(B)** CC and nRMSE presented as functions of relative catheter volume for the 64-electrode catheter (blue) and 130-electrode catheter (red). Median values and IQR are given. Abbreviations: MFS, Method of Fundamental Solutions; LA, left atrium; CC, correlation coefficient; nRMSE, normalized root-mean-squared error; IQR, interquartile range.

While potential maps during macro reentrant activity were reconstructed more faithfully using a 130-electrode catheter than a 64-electrode catheter with the same dimension, corresponding phase maps in Figure 5A appear to carry very similar information about the history of activation across the LA surface. This similarity in phase distribution was preserved throughout an extended sequence of simulated electrical activity (Supplementary Video S1), where phase singularities identified recovered with 64- and 130-electrode catheters are also collocated. The correspondence with ground truth for phase maps obtained with noncontact catheters was maintained for a wider range of relative catheter volumes than for the potential maps in Figure 3 above. However, CC was increased and nRMSE reduced with a 130-electrode catheter compared to a 64-electrode catheter (Figure 5B). This indicates that phase maps with the latter capture key features of wave front propagation in macro reentrant arrhythmia, but aspects of the fine structure of the phase distribution are lost.

### Region-Of-Interest Potential Mapping

**FIGURE 6:**
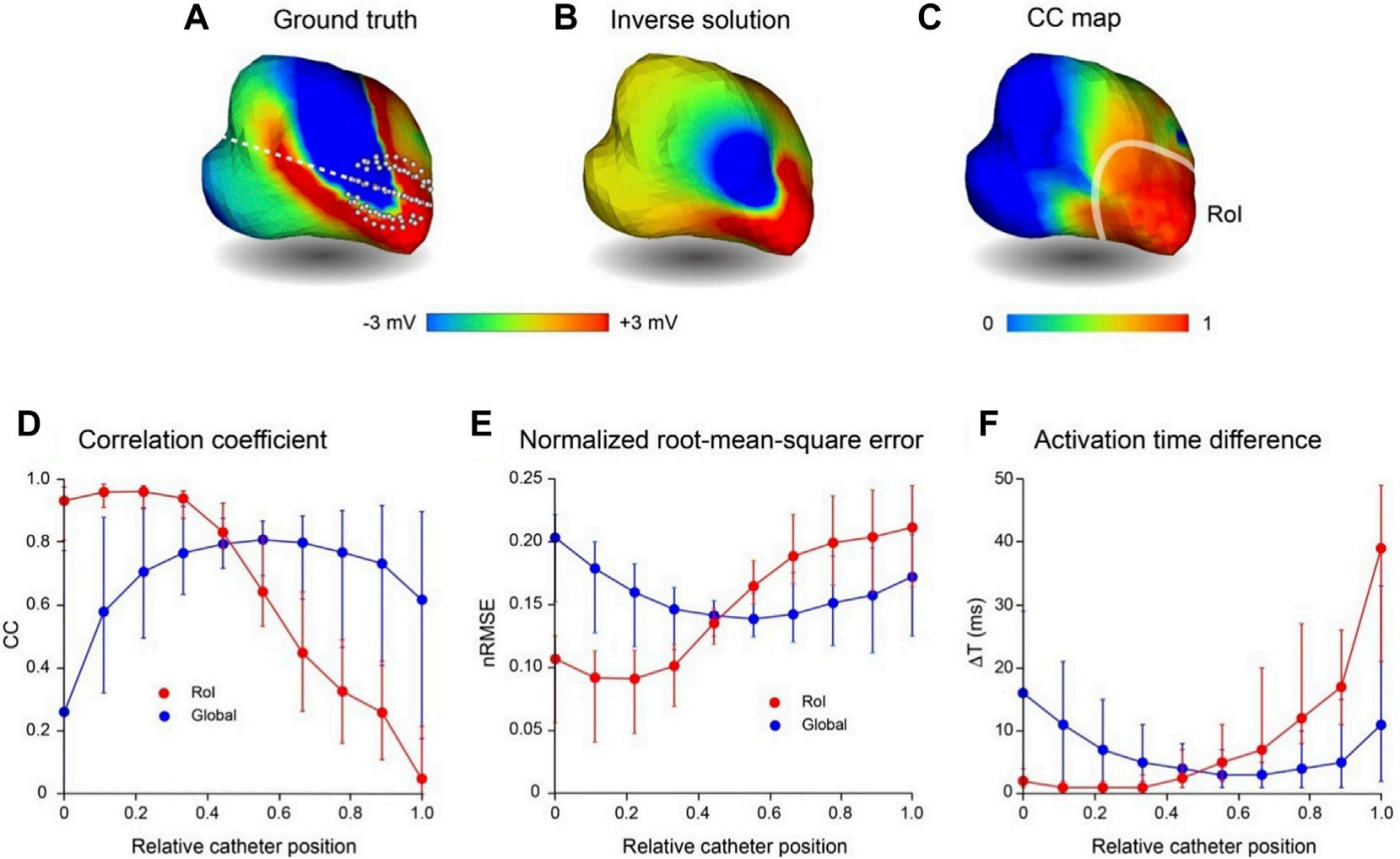
Comparison of region-of-interest inverse potential mapping with global mapping. LA surface potentials during 3 activation cycles of simulated macroscopic reentry were reconstructed from electrograms sampled inside the LA cavity with a small 64-electrode basket catheter. The catheter was initially positioned with some electrodes touching the wall at the junction with the LAA and then moved rightward until electrodes made contact with the inter-atrial septum. **(A)** Representative ground truth potentials on cavity surface also showing broken white line along which catheter is moved from origin of LAA (relative catheter position 0) to inter-atrial septum (relative catheter position 1). **(B)** Inverse solution reconstructed from potentials sampled at relative catheter position 0. **(C)** Correlation coefficient map for ground-truth and inverse solutions in this case. The RoI indicated is the smoothed boundary of the surface in **(C)** where CC ≥ 0.9. In the lower panel **(D–F)** present CC, nRMSE and ΔT, respectively, in the RoI (red) and across the complete LA surface (blue) for inverse solutions with the catheter at equi-spaced locations along the line in **(A)**; catheter locations 0 and 1 are indicated in **(A)**. Median values and interquartile range (IQR) are given in lower panel. Abbreviations: RoI, region of interest; LA, left atrium; LAA, left atrial appendage; CC, correlation coefficient; nRMSE, normalized.

With region-of-interest (RoI) mapping, a small catheter is positioned close to a region of the endocardial surface to reconstruct local electrical activity. In Figure 6, we consider the accuracy with which regional electrical activity can be recovered using non-contact catheters. This analysis was completed without adding Gaussian noise. An 8-spline 64-electrode basket catheter (major and minor axes 25 and 23 mm, respectively) was initially located close to the origin of a simulated macro-reentrant circuit in the LA (Figure 6A). The inverse solution (Figure 6B) was good near the catheter, but much poorer over the rest of the LA. This is demonstrated in Figure 6C where CC is rendered on the LA surface; median CC is >0.9 in the RoI, but falls off rapidly with distance from this region. In the lower panel we present CC (Figure 6D), nRMSE (Figure 6E) and ΔT (Figure 6F) in the RoI (red) and across the full endocardial surface (blue) for inverse solutions constructed as the catheter was moved progressively along a line from the origin of the LAA to the inter-atrial septum (Figure 6A). These figures demonstrate that regional mapping performance was excellent when the catheter was in or adjacent to the RoI, but poor when the catheter was most distant from it. Global mapping performance was best when the catheter was located centrally, but significantly poorer in this case in the RoI. Of particular interest, RoI performance was optimal when the catheter was ∼10 mm from its initial position with electrodes 9–20 mm from the LA wall; median CC was 0.96 [IQR 0.072], median nRMSE 0.09 [IQR 0.05] and median ΔT 0.89 ms [IQR 1.97 ms].

## Discussion

### Summary

This analysis of noncontact intracardiac potential mapping extends an *in-silico* boundary mesh-based study previously reported by our laboratory (Meng et al., 2017; Meng et al., 2022). Here we have investigated the accuracy with which endocardial potential maps can be reconstructed from noncontact electrograms recorded with a multi-electrode basket catheter using meshless methods that use the MFS, the first time that this has been done as far as we are aware. We demonstrate that fast, accurate noncontact potential mapping and phase mapping are possible using this approach. However, the spatial frequency of the electrical activity captured is determined by the distribution of electrodes and in order to recover complex non-stationary rhythms, such as AF, the mapping catheter must address a representative subvolume of the cardiac chamber.

### Effects of Catheter Dimension

We have reported that noncontact mapping performance deteriorates progressively as catheter dimensions are reduced relative to those of the cardiac chamber and that this degradation becomes more marked when activation is complex. Neither of these findings is surprising. With increasing distance from the heart surface, intracardiac potentials associated with local activation are progressively attenuated and blurred, and information lost in this process cannot be recovered fully. Furthermore, as catheter dimension is reduced, information is captured from a decreasing subset of the cavity volume which may not fully reflect local activity. More striking, perhaps, is the finding that surface electrograms can be reconstructed with acceptable accuracy (CC > 0.9, nRMSE ≤0.06 and ΔT ≤ 2 ms) during reentrant rhythm with basket catheters that fill only half of the cavity. A supplementary point that needs to be made here, is that while relative catheter volume is an accessible measure of dimension, it scales with the third power of radius for a spherical basket catheter. Therefore, catheter volume increases by 112.5% when its diameter changes from 35 to 45 mm. While there was no contact between electrodes and LA wall for the centrally located catheter in Figure 3A (Catheter:Atrial volume ratio = 0.67), there was increasing (though incomplete) contact between them as relative volume expanded to ∼0.9.

### Effects of Noise

Median CC was decreased and median ΔT was increased with reduced catheter dimension (Figures 4B,D) when Gaussian noise was added but there was no corresponding effect on median nRMSE (Figure 4C). The reconstructed electrograms in Figure 4A provide explanation for these results. Because electrograms recorded toward the centre of the LA cavity with a small central catheter are attenuated, the noise added to them markedly reduces SNR. This is reflected in the reconstructed surface electrograms presented in the left-hand panel of Figure 4A, where SNR is low and is reduced progressively as noise amplitude increases. The recorded electrograms are also smoothed in this case and the frequency content of the ground-truth surface electrograms is not recovered by inverse mapping. However, the overlap between ground-truth and reconstructed electrograms is affected less by noise than might have been expected. With increased noise power, the deviation between reconstructed and ground-truth electrograms can increase, but there is also greater instantaneous overlap between the two. With a large catheter (right-hand panel in Figure 4A), the magnitudes of recorded electrograms are substantially greater and there is much less smoothing. As a result, surface electrograms are recovered more faithfully with much less impact of added noise.

Inverse methods are prone to instability and error in the presence of noise. The fact that this is not the case here further reinforces the fact that the transfer matrix used is inherently well-conditioned and the regularization procedures adopted are appropriate. However, the Gaussian noise introduced here is a very narrow representation of the problems faced in practice with inverse potential mapping. Artifacts in the unipolar signals used for this purpose include common-mode electrical noise, far-field activity due to electrical activation of the ventricles which can mask local activity completely in AF and complexity in atrial electrograms that may be due to far-field atrial activity, inadequate spatial sampling or non-uniform electrical properties in the underlying substrate. That said there are many ways that more robust regional information can be extracted from these channels using signal processing methods that exploit temporal and spatial correlation among adjacent electrograms and wavelet-based methods which identify characteristic differences in the instantaneous frequency content of recorded electrograms (Zhao et al., 2013).

### Electrode Distribution and Recovery of Complex Activation Patterns

Our data show that ground truth potential maps based on simulated macro-reentrant activity were reconstructed more faithfully using a 130-electrode catheter than a 64-electrode catheter with the same dimension (Figure 3). Furthermore, when noncontact electrodes were within a few mm of the cavity surface, dimension had no effect on the efficacy of the inverse solution, which was wholly dependent on electrode distribution. This reflects the fact that the accuracy with which surface potentials can be reconstructed depends on whether the sampled potentials provide a faithful representation of the field addressed. If the electrode distribution is not sufficiently dense, high spatial frequencies cannot be recovered and low frequency artifacts (aliasing) may occur (Shannon, 1949). An example of this is provided in Figure 2 where apparent fractionation of the electrogram reconstructed between adjacent splines with a 64 electrode basket catheter (see blue trace in Figure 2F) disappears with more dense sampling in that region (compare red trace with ground truth electrogram in Figure 2F). While compressed sensing approaches can collect and represent sparse signals with many fewer sampling points than indicated by the Nyquist (Shannon, 1949) theorem, optimal sampling strategies are determined by regional spatial and temporal correlation (Long et al., 2011). Specialized regularization techniques are also needed for inverse reconstruction of higher frequencies from sparsely sampled signals without introducing excessive noise.

It should be noted that metrics such as CC and nRMSE used here to quantify the correspondence between ground-truth and reconstructed potentials do not take the time-history of the electrograms into account. This provides additional information about the spread of electrical activation across the heart surface as illustrated by the phase maps in Figure 5. The phase map shown here for a 64-electrode catheter is very similar to that presented for a 130-electrode catheter. Both correspond closely to ground truth phase maps throughout the activation sequence (Supplementary Video S1). Because phase mapping identifies local activation as an abrupt standardized transition from +π to −π and imposes relatively uniform spatio-temporal variation around this, confounding effects of local variation in potential magnitude are removed.

It is also important to acknowledge that sampling density is affected by catheter dimensions. For instance, for a 64-electrode 25-mm diameter spherical catheter, inter-electrode spacing along splines is ∼5 mm, while the curvilinear distance between splines at the equator is ∼10 mm. These measurements are doubled with a 50-mm diameter catheter. This explains the apparent reduction in median CC and the increase in median nRMSE and its interquartile range for 64-electrode catheters when relative catheter volume increases from 0.8–0.91 (Figures 3D,E, respectively). Here any improvement in accuracy associated with proximity to the wall is offset by reduced electrode density. In the clinical setting, attempts to achieve direct contact between electrodes and the heart surface can introduce additional error by deforming catheter splines and increasing the nonuniformity of sampling. (Oesterlein et al., 2016; Pathik et al., 2018). It follows that global mapping with multi-electrode basket catheters is more likely to produce reliable results when electrodes are not in contact with the heart wall than when attempts are made to achieve close contact.

The results for RoI mapping are consistent with these observations. The relatively small 64-electrode catheter in Figure 6 recovered local electrical activity with a high level of accuracy. Moreover, regional mapping produced best results when electrodes were not in contact with the wall (median CC, nRMSE and ΔT: 0.96, 0.09 and 0.89 ms, respectively). Global performance was much poorer, but it would be straightforward to quantify the uncertainty of reconstructed maps based on the distance of electrodes from surface nodes and the results of analyses such as those outlined here.

### Potential Clinical Impact of These Findings

The results of this study indicate that global electroanatomic maps can be recovered faithfully in real-time from electrograms recorded with noncontact multi-electrode basket catheters using meshless methods that use the MFS. Accurate specification of 3D electrode locations with respect to cardiac anatomy is required for inverse intracardiac mapping, but this is readily achieved with current hybrid navigation technologies (Issa et al., 2019). Our findings indicate that, for optimal performance, catheters should be located centrally within the cardiac chamber and address a representative subvolume of the cavity (>50% in the data presented here) with minimum contact between electrodes and endocardial surface. The capacity to reconstruct spatially complex activation patterns is limited by electrode distribution, but when heart rhythm is stable and repeated, more detailed maps can be reconstructed with sequential alteration of electrode locations, for instance by catheter rotation/translation. Potentially, this could be more efficient than sequential contact mapping with high density contact arrays because complete maps can be developed with relatively few iterations. For nonstationary heart rhythms such as AF, however, the accuracy with which endocardial surface activation can be reconstructed is constrained by the spatial distribution of electrodes on the catheter for both contact and noncontact mapping. Sparse sampling can lead to repeating artifact in reconstructed activation patterns that is incorrectly identified as rotors (Roney et al., 2017; Martinez- Mateu et al., 2018). Williams et al. (Williams et al., 2018) reported that >1.0–1.5 points/cm^2^ were needed on the endocardial surface to resolve spiral wave activity and this corresponds to an inter-electrode spacing of 2–3 mm - much denser than is the case for 64-electrode basket catheters, particularly for equatorial electrodes on adjacent splines. As demonstrated here, more optimal electrode distribution is achieved with catheters that have 16 rather than 8 splines. While phase mapping may relax sampling requirements to some extent, it seems evident that improved catheter design is necessary for accurate panoramic mapping in AF.

Inverse methods have been used for noncontact intracardiac electrical mapping in two commercial systems. (Schilling et al., 1999; Grace et al., 2019). The Ensite multi-electrode array (Abbott) is used for noncontact potential mapping and consists of 64 electrodes mounted on an inflatable balloon. Consistent with the results reported here, validation studies have shown that accuracy is inversely related to the distance between the array and the heart wall with poor recovery of endocardial surface potentials when this distance is >25 mm (Earley et al., 2006). More recently, instantaneous charge density distributions associated with atrial electrical activation have been constructed from electrograms recorded with noncontact 48-electrode basket catheters (Acutus/Biotronik) (Grace et al., 2019). This is based on a forward model that relates intracardiac potential fields to secondary cellular sources associated with distributed membrane charge dipoles (Plonsey, 1982; Grace et al., 2019; Willems et al., 2019). Our analysis makes no assumptions about the cellular basis of electrical activation. Instead, we address how well endocardial surface potentials can be recovered from a limited number of electrograms recorded inside the heart cavity. We found that information is lost with noncontact mapping if the basket catheters used are too small and with both contact and noncontact mapping if the sampling density is not sufficient. These factors would be expected to impact the spatial resolution with which surface charge distributions can be recovered from noncontact electrograms also.

### Limitations

A limitation of this study is that error introduced by uncertainty of the 3D geometry of the heart surface relative to 3D electrode locations has not been explicitly considered. While this would be expected to amplify uncertainty associated with relative catheter size, electrode distribution and noise, we note that our formulation of the intracardiac inverse problem is surprisingly robust. A further limitation is that although our ground-truth data represent atrial rhythms of increasing complexity, they do not fully replicate the spatio-temporal disorder which characterizes AF. However, the analysis presented here demonstrates that the performance of contact mapping is matched by this inverse approach and that spatial resolution in both cases is limited by electrode distribution. Finally, while ground-truth data are based in part on clinical and simulated data, the accuracy of inverse intracardiac mapping has been confirmed computationally. While many of the assumptions made in specifying the forward problem are entirely reasonable, other are not. The electrical properties of the blood in the cardiac chambers are isotropic and uniform but they are certainly not the same as those in the myocardium adjacent to the endocardial surface where the fictitious sources are located. More detailed experimental characterization of the accuracy with which endocardial potentials can be reconstructed using inverse mapping is therefore needed to confirm the analyses presented here.

## Conclusion

This study demonstrates that atrial endocardial potentials can be reconstructed accurately from electrograms recorded with noncontact multi-electrode basket catheters using a fast robust inverse mapping approach that employs the MFS. This enables efficient and potentially more precise capture of global and region-of-interest potential maps than comparable contact mapping methods. Because data for all electrodes are used, it is not necessary to maximize contact between catheter and the heart wall. This reduces the deformation of catheter splines which occurs when direct contact is sought, thereby preserving a more uniform electrode distribution. However, we demonstrate that conventional 8 spline catheters are suboptimal for instantaneous contact or noncontact mapping of complex rhythms, such as AF.

## Data Availability

The raw data supporting the conclusion of this article will be made available by the authors, without undue reservation.
