# Supplementary figures and images for: Intracardiac Inverse Potential Mapping Using the Method of Fundamental Solutions
Source: Front Physiol. 2022 May 16;13:873049. doi: 10.3389/fphys.2022.873049 (PMC9149204; doi:10.3389/fphys.2022.873049)

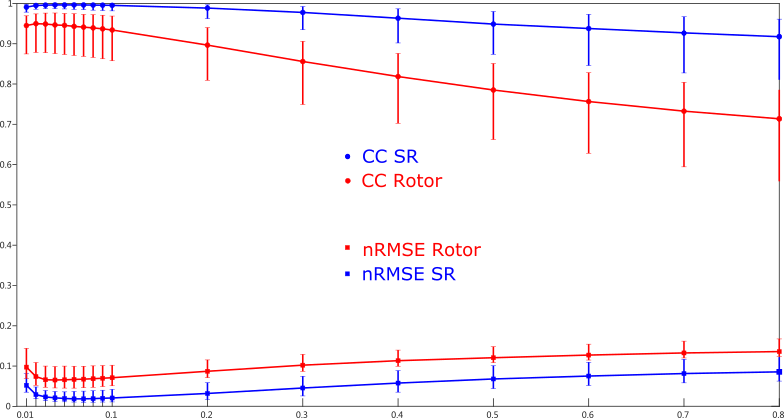

Supplement: Supplementary file 1 [file Image1.PNG]
